# *N*-Caffeoyltryptamine, a Potent Anti-Inflammatory Phenolic Amide, Suppressed MCP-1 Expression in LPS-stimulated THP-1 Cells and Rats Fed a High-Fat Diet

**DOI:** 10.3390/ijms18061148

**Published:** 2017-05-27

**Authors:** Jae B. Park

**Affiliations:** Diet, Genomics, and Immunology Laboratory, Beltsville Human Nutrition Research Center, The Agricultural Research Service, The United States Department of Agriculture, Bldg. 307C, Rm. 131, Beltsville, MD 20705, USA; jae.park@ars.usda.gov; Tel.: +1-301-504-8365; Fax: +1-301-504-9062

**Keywords:** *N*-caffeoyltryptamine, p38 MAP kinase, MCP-1, high-fat diet, rats

## Abstract

Monocyte chemoattractant protein-1 (MCP-1) is a well-known chemokine critically involved in the pathophysiological progression of several inflammatory diseases including arthrosclerosis. *N*-caffeoyltryptamine is a phenolic amide with strong anti-inflammatory effects. Therefore, in this paper, the potential effect of *N*-caffeoyltryptamine on MCP-1 expression was investigated as a potential p38 mitogen-activated protein (MAP) kinase inhibitor in vitro and in vivo. At the concentration of 20 μM, *N*-caffeoyltryptamine significantly inhibited p38 MAP kinase α, β, γ and δ by 15–50% (*p* < 0.05), particularly p38 MAP kinase α (IC_50_ = 16.7 μM) and β (IC_50_ = 18.3 μM). Also, the pretreatment of the lipopolysaccharide (LPS)-stimulated THP-1 cells with *N*-caffeoyltryptamine (10, 20 and 40 μM) led to significant suppression of MCP-1 production by 10–45% (*p* < 0.05) in the cells. Additionally, *N*-caffeoyltryptamine was also able to significantly downregulate MCP-1 mRNA expression in the THP-1 cells (*p* < 0.05). On the basis of this strong inhibition in vitro, an animal study was conducted to confirm this inhibitory effect in vivo. Rats were divided into three groups (*n* = 8): a normal control diet (C), a high-fat diet (HF), or a high-fat diet supplemented with *N*-caffeoyltryptamine (2 mg per day) (HFS). After 16 weeks, blood samples were collected from the rats in each group, and MCP-1 levels were determined in plasma with other atherogenic markers (C-reactive protein and soluble E-selectin (sE-selectin)). As expected, the average MCP-1 levels of the HF group were found to be higher than those of the C group (*p* < 0.05). However, the MCP-1 levels of the HFS group were significantly lower than those of the HF group (*p* < 0.05), suggesting that *N*-caffeoyltryptamine could decrease MCP-1 expression in vivo. Related to other atherogenic markers such as C-reactive protein and sE-selectin, there was no significant difference in their levels between the HF and HFS groups. These data suggest that *N*-caffeoyltryptamine may specifically suppress MCP-1 expression in vitro and in vivo, possibly by inhibiting p38 MAP kinase.

## 1. Introduction

Cardiovascular disease (CVD) is still one of major causes for human mortality worldwide [1]. A great number of studies suggest that chronic cardiovascular inflammation may be a significant contributing factor in the initiation and development of atherosclerotic CVD [1,2,3,4,5]. Monocyte chemoattractant protein 1 (MCP-1, or CCL2) is a potent mononuclear chemokine, which can regulate monocyte and macrophage migration and infiltration [6,7]. Due to its strong chemotactic activity, MCP-1 has the ability to recruit monocytes into inflamed blood vessel walls, which is one of the earliest events in atherosclerosis, and eventually advance the progression of plaque instability and vascular injury [8]. In humans, MCP-1 has been detected in atherosclerotic plaques [9], and MCP-1 plasma concentrations have been found to be relatively high in patients with CVD risk factors [10], suggesting that MCP-1 may play a critical role at multiple stages of atherosclerosis [11,12].

*N*-caffeoyltryptamine (safflomide), *N*-coumaroyltryptamine and *N*-feruloyltryptamine are phenolic amides belonging to a group of tryptophan-derived phenylpropenoid amides found in plants such as *Carthamus tinctorius*, *Centaurea cyanus*, *Coffea* sp., *Ipomoea obscura*, and *Theobroma cacao* [13,14,15,16]. Especially, *N*-coumaroyltryptamine was found in *Carthamus tinctorius* and *Zea mays* [13,16]. However, there is little information about their quantities in human diet components, although their potential biological activities have been investigated for years. Interestingly, our studies showed that *N*-caffeoyltryptamine has stronger antioxidant, anti-inflammatory and adiponectin-increasing activities than *N*-coumaroyltryptamine and *N*-feruloyltryptamine [17,18], suggesting that *N*-caffeoyltryptamine could be a better candidate and potentially used as a bioactive supplementation related to human health. Therefore, in this study, the potential effects of *N*-caffeoyltryptamine on p38 MAP kinase and MCP-1 expression were investigated, because p38 MAP kinase plays a vital role in regulating the biosynthesis of many inflammatory cytokines including MCP-1 [19,20]. First, the potential effects of *N*-caffeoyltryptamine on the isoforms (p38α, p38β, p38γ, p38δ) of p38 MAP kinase were investigated in vitro. Then, the effects of *N*-caffeoyltryptamine on MCP-1 mRNA and protein expression were studied in the lipopolysaccharide (LPS)-stimulated THP-1 cells. Afterward, an animal study was conducted using rats fed a high-fat diet in order to determine its effects on MCP-1 protein in vivo. Additionally, the potential effects of *N*-caffeoyltryptamine on C-reactive protein and sE-selectin were examined in plasma, because they are also considered as good atherosclerotic markers for screening several CVDs [21,22].

## 2. Results

### 2.1. Effects of N-Caffeoyltryptamine on p38 MAP Kinase Activity

**Figure 1:**
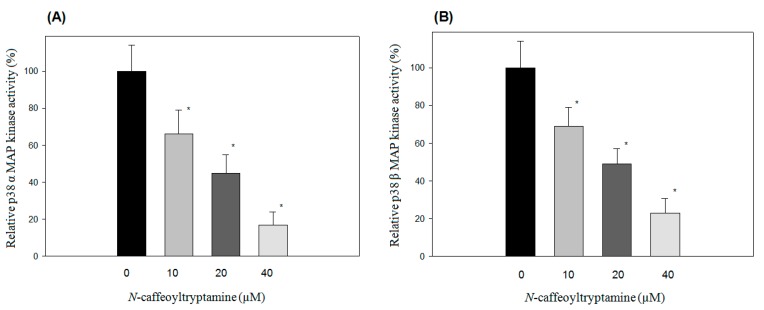

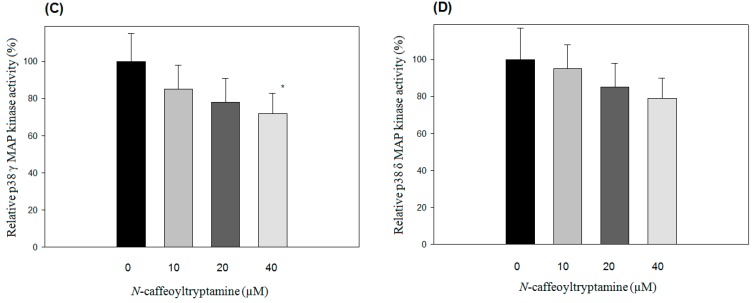
Inhibition of p38 MAP kinase isoforms by *N*-caffeoyltryptamine. The inhibition of p38 α (**A**), β (**B**), γ (**C**) and δ (**D**) was determined at 10, 20, and 40 µM. Data points are shown with the mean ± standard deviation (SD) (*n* = 5). *p* Value was calculated using one-way ANOVA with the Holm—Sidak method and the marks (*) indicate statistical significance (*p* < 0.05). In (**A**,**B**), the tested groups were statistically significant compared to the control and each group; In (**C**), only the group (40 µM) was statistically significant compared to the control.

The p38 mitogen-activated protein kinase (MAPK) pathway is deeply involved in the production of inflammatory chemokine including MCP-1 [19,23]. In this study, the effects of *N*-caffeoyltryptamine on p38 MAP kinase isoforms (p38α, β, γ (ERK6 or SAPK3) and δ (SAPK4)) were investigated using the CMGC-1 kinase selectivity profiling system (Promega, Madison, WI, USA), as described in “Section 4”. As shown in Figure 1, *N*-caffeoyltryptamine was able to inhibit all p38α (Figure 1A), β (Figure 1B), γ (Figure 1C) and δ (Figure 1D) to some extent, especially p38α and p38β; the decreasing order of the inhibition was p38β ˃ p38α ˃ p38γ ≥ p38δ. These data suggested that *N*-caffeoyltryptamine could inhibit p38 MAP kinases (especially α and β forms).

### 2.2. IC_50_ of p38 MAP Kinase α and β Inhibition

**Figure 2:**
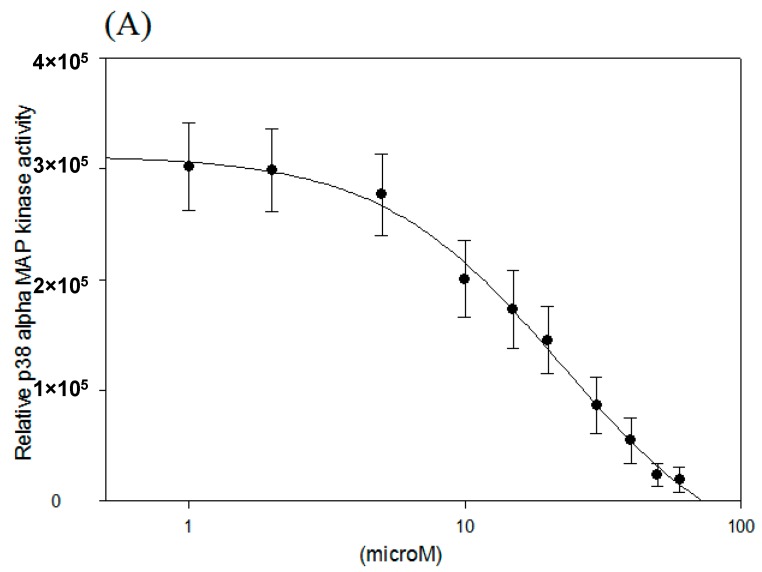

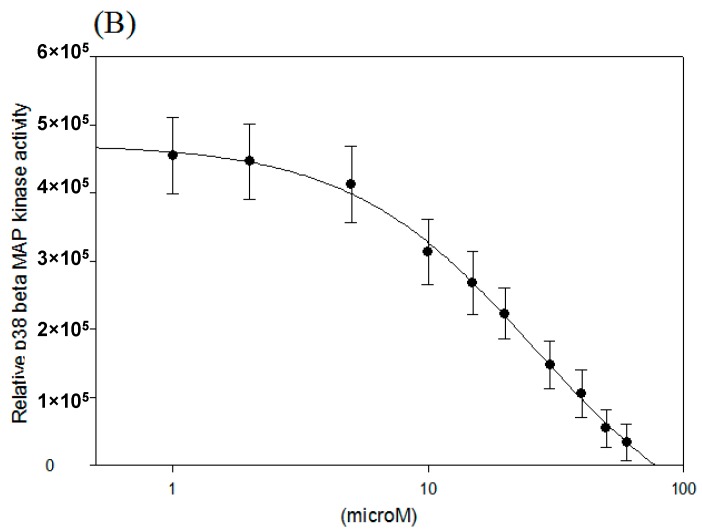
IC_50_ of p38 MAP kinase α and β. The inhibition of p38 α (**A**) and β (**B**) was determined at the range between 0 and 60 µM. Data points are shown with the mean ± SD (*n* = 3).

Since *N*-caffeoyltryptamine was able to inhibit p38α and β to a greater degree than it inhibited p38γ and δ, the IC_50_ for p38α and p38β was exclusively determined at the concentrations between 0–60 μM. As shown in Figure 2A,B, the inhibition was almost complete at the concentrations higher than 60 μM, and the IC_50_ values for p38 MAP kinase α and β were found to be 16.7 and 18.3 μM, respectively. In fact, p38 MAP kinase α and β are two well-studied isoforms that are believed to be critically involved in cytokine production and inflammatory process [19]. Therefore, it is likely that *N*-caffeoyltryptamine inhibits p38 MAP kinase α and β, thereby potentially affecting downstream molecules involved in the transcription of MCP-1 in the cells as described next.

### 2.3. Effect of N-Caffeoyltryptamine on MCP-1 mRNA in THP-1 Cells

**Figure 3:**
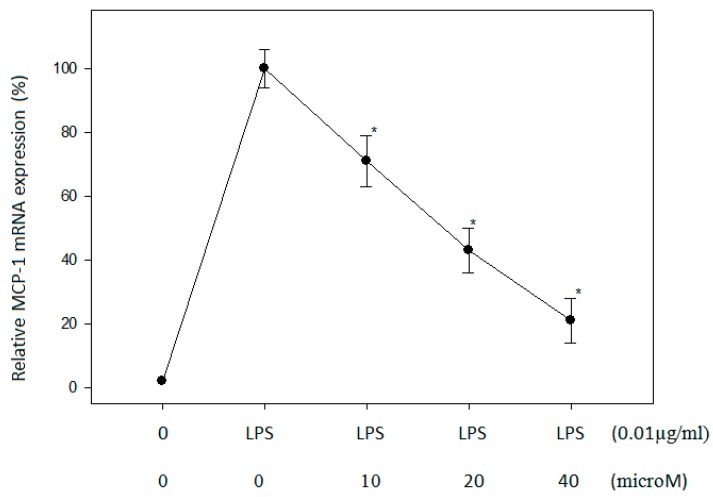
Effects of *N*-caffeoyltryptamine on MCP-1 mRNA in the lipopolysaccharide (LPS)-stimulated THP-1 cells. THP-1 cells were treated with 0, 20 and 40 µM *N*-caffeoyltryptamine, RNA was isolated at 4 h, and gene expression of MCP-1 was determined using real-time polymerase chain reaction PCR. Results are expressed as % inhibitory effect means ± SD (*n* = 3). Data were analyzed using one-way ANOVA with the Holm—Sidak method as described in the “Section 4”. (*) depicted significant difference compared to the control and each group (*p* < 0.05).

Due to the ability of *N*-caffeoyltryptamine to inhibit the p38 MAP kinases (Figure 2), the dose-dependent effects of the compound on MCP-1 mRNA expression were investigated in the THP-1 cells. The cells were treated with different concentrations of *N*-caffeoyltryptamine (0, 10, 20, and 40 µM), and MCP-1 mRNA expression was compared as described in “Section 4”. As shown in Figure 3, *N*-caffeoyltryptamine could decrease MCP-1 mRNA expression in a dose-dependent manner. These data suggest that *N*-caffeoyltryptamine was capable of suppressing MCP-1 mRNA expression, thereby reducing MCP-1 protein expression in the cells.

### 2.4. Effect of N-Caffeoyltryptamine on the MCP-1 Protein in THP-1 Cells

**Figure 4:**
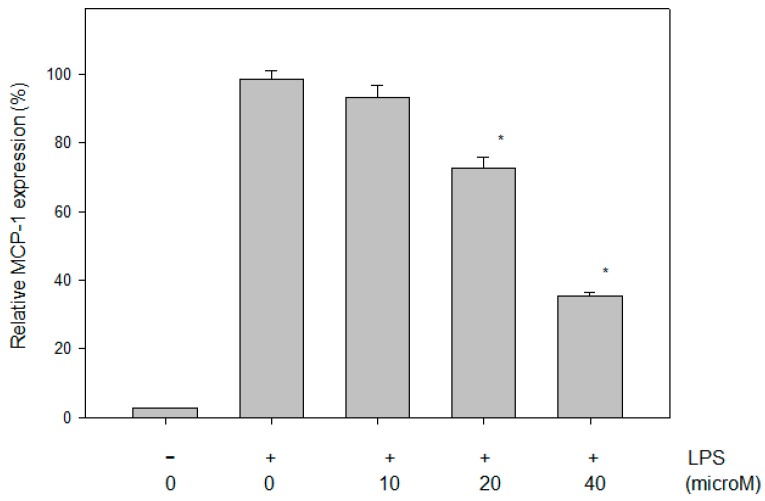
MCP-1 inhibition by *N*-caffeoyltryptamine in the LPS-stimulated THP-1 cells. The cells were incubated for 15 min with several concentrations of *N*-caffeoyltryptamine (0, 10, 20, 40 µM), then treated with LPS (0.01 µg/mL) for 16 h. MCP-1 assay was performed as described in “Section 4”. Data points are shown with the mean ± SD (*n* = 5). *p* Value was calculated using one-way ANOVA with the Holm—Sidak method and the marks (*) indicate statistical significance compared to control group (*p* < 0.05).

Because *N*-caffeoyltryptamine inhibited p38 MAP kinases (Figure 2) as well as MCP-1 mRNA expression (Figure 3), and because several studies suggested that the inhibition of p38 MAPK could lead to the suppression of the production of MCP-1 in the cells [8,9], the effects of the compounds on MCP-1 expression were investigated in monocytic THP-1 cells stimulated with LPS. The LPS treatment induced MCP-1 protein significantly in the THP-1 cells, and the increased MCP-1 protein was greatly suppressed by the treatment with *N*-caffeoyltryptamine (10, 20 and 40 µM) (Figure 4). The data demonstrated that the treatment of the THP-1 cells with *N*-caffeoyltryptamine (10–40 µM) could lead to significant inhibition (*p* < 0.05) (Figure 4).

### 2.5. In Vivo Effects of N-Caffeoyltryptamine on MCP-1 Expression

**Figure 5:**
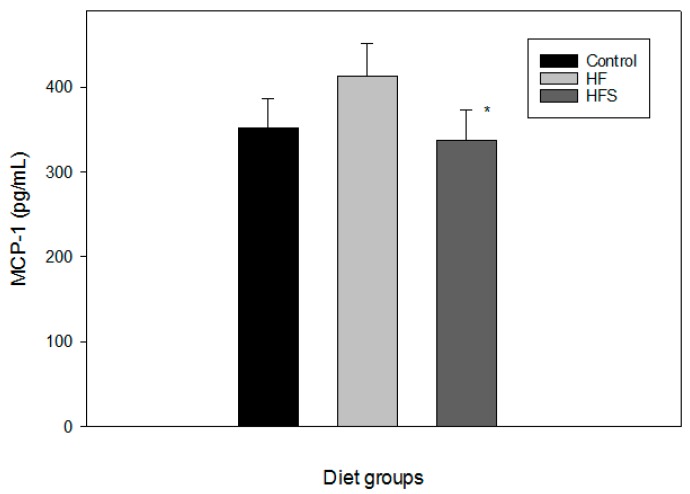
Effects of *N*-caffeoyltryptamine on MCP-1 in rats. Plasma MCP-1 was measured as described in “Section 4”. C, normal control diet; HF, high-fat diet; HFS, high-fat diet supplemented with *N*-caffeoyltryptamine (2 mg per day). Data are presented as the mean ± SD (*n* = 8). *p* Value was calculated using one-way ANOVA with the Holm—Sidak method and the marks (*) indicate statistical significance compared to HF group (*p* < 0.05).

The data in this study clearly indicate that *N*-caffeoyltryptamine is a potential inhibitor of p38 MAP kinases, possibly suppressing MCP-1 mRNA and protein expression in the THP-1 cells. The next step was to determine whether *N*-caffeoyltryptamine could inhibit MCP-1 expression in vivo. To address this question, an animal study was conducted using a rat model. In this study, rats were divided into three groups (C, HF, and HFS) and fed C, HF, and HFS diets (Table 1) for 16 weeks. The initial body weights and feed intakes of the rats in the three groups were not significantly different (Table 2). The average body weight of the rats in the HF group was higher than that of the rats fed the C diet, and the bodyweight of rats fed the HFS diet was slightly lower than that of the rats fed the HF. However, the difference was not statistically significant (Table 2). At the end of 16 weeks, blood was collected for the assays as described in “Section 4”. Since the difference of the body weights of HF and HFS groups were insignificant compared to the control group, no significant difference was observed in liver and visceral fat weights. Furthermore, the difference in the levels of plasma cholesterol, low-density lipoprotein cholesterol (LDL), high-density lipoprotein cholesterol (HDL) and triglyceride (TG) in rats in all three groups were not significant either (Table 3). However, rats fed a HF diet demonstrated increased plasma levels of MCP-1 by more than 10% compared to rats on the C diet (Figure 5), and the rats fed the HFS diet (supplemented with *N*-caffeoyltryptamine (2 mg per day)) demonstrated significantly lower plasma MCP-1 levels than those fed the HF diet (*p* < 0.05), and even slightly lower than those on the control diet (Figure 5). Because plasma levels of MCP-1 are commonly used as a risk indicator for CVD including atherosclerosis [11,12], and because a high-calorie diet often increases plasma levels of the MCP-1 protein in humans [24], these data suggest that *N*-caffeoyltryptamine may have positive effects in lowering the plasma levels of MCP-1 in individuals with cardiovascular risks fed a high-calorie diet.

### 2.6. Effects of N-Caffeoyltryptamine on Plasma C-Reactive Protein

**Figure 6:**
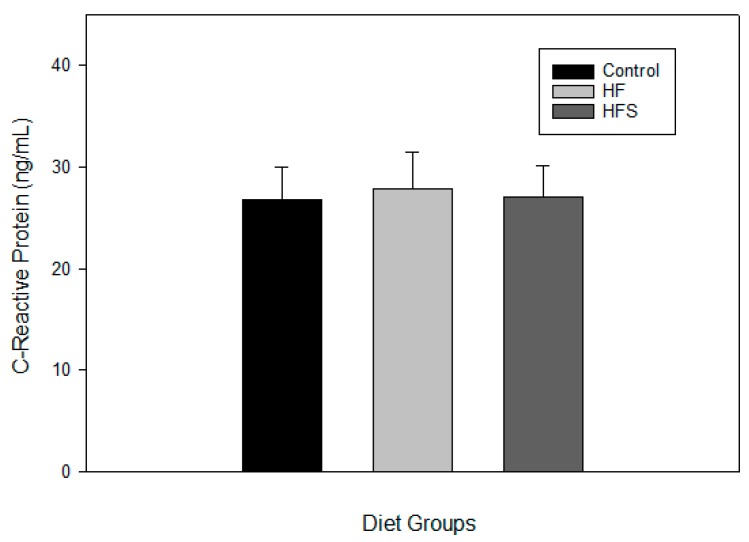
Effects of *N*-caffeoyltryptamine on C-reactive protein in rats. Plasma C-reactive protein was measured as described in “Section 4”. C, normal control diet; HF, high-fat diet; HFS, high-fat diet supplemented with *N*-caffeoyltryptamine (2 mg per day). Data are presented as the mean ± SD (*n* = 8).

Although the data suggest that the supplementation of *N*-caffeoyltryptamine may suppress plasma MCP-1 levels via inhibition of the p38 MAP kinases in rats fed the HFS diet, the effect of *N*-caffeoyltryptamine could be one of the events manifested by lowering the overall risk factors for CVD that is not related to p38 MAP kinases. Therefore, the effects of *N*-caffeoyltryptamine on the plasma levels of another atherosclerotic risk factor (C-reactive protein (CRP)) were investigated in rats, because several reports also suggested that this protein is also a reliable atherosclerotic risk factor in patients with progressed CVD [25,26,27,28,29]. As shown in Figure 6, the mean values of plasma CRP in the HFS group were lower than those in the HF group, but the differences between the groups were not statistically significant (C, HF, and HFS). These data suggest that the effect of *N*-caffeoyltryptamine as a potential p38 MAP kinase inhibitor may not generate a significant difference in the plasma levels of CRP in rats, which agrees with previous studies where p38 MAP kinase inhibitors did not reduce CRP compared to the placebo in humans [25,26].

### 2.7. Effects of N-Caffeoyltryptamine on Plasma sE-Selectin

**Figure 7:**
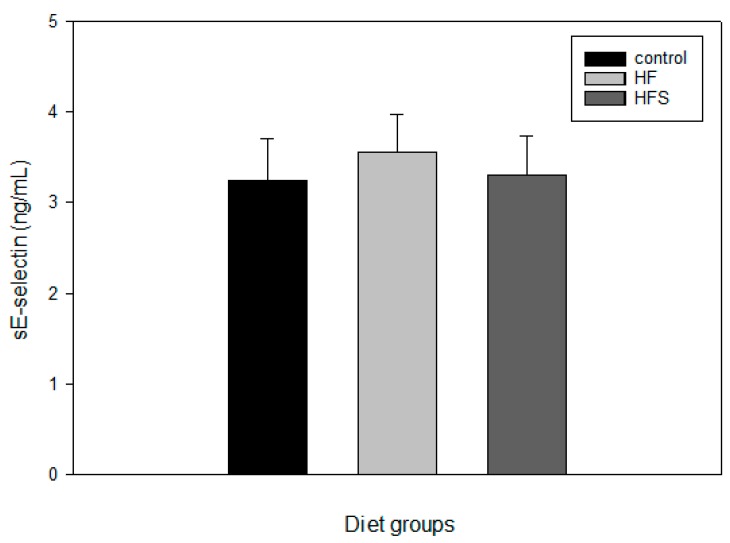
Effects of *N*-caffeoyltryptamine on sE-selectin in rats. Plasma sE-selectin was measured described in “Section 4”. C, normal control diet; HF, high-fat diet; HFS, high-fat diet supplemented with *N*-caffeoyltryptamine (2 mg per day). Data are presented as the mean ± SD (*n* = 8).

Additionally, the effects of *N*-caffeoyltryptamine on plasma sE-selectin were investigated using the same blood samples, because this protein is also considered a reliable atherosclerotic risk factor [29], As with the CRP, the supplementation provided no significant difference between the C, HF, and HFS groups (Figure 7), although the mean values of plasma sE-selectin were slightly different in the HF and HFS groups. These data suggest that the supplementation with *N*-caffeoyltryptamine may not provide significant difference in plasma levels of CRP and sE-selectin in rats between the C, HF, and HFS groups. All these data suggest that the supplementation with *N*-caffeoyltryptamine may to a certain extent specifically lower plasma levels of the MCP-1 expression in rats fed a HF diet, which may be closely associated with the pathway of p38 MAP kinase.

## 3. Discussion

CVD is one of the major causes of human mortality, and chronic cardiovascular inflammation is believed to play a significant causative role in initiating and developing CVD, especially atherosclerosis [1,2,3,4,5]. Among the inflammatory chemokines involved in the process of atherosclerosis, MCP-1 (CCL2) is considered a potent culprit that is involved in the earliest events, progression and vascular injury in this disease because MCP-1 has strong chemotactic activity to recruit monocytes to inflamed blood vessel walls [6,7]. Several studies using p38 MAPK inhibitors suggest a crucial role of p38 MAPK in the progression of acute/chronic inflammation in CVD [25,26,27,28,29,30]. However, studies did not always support all beneficial effects of the inhibitors on acute/chronic inflammation in CVD and other CVD-related disease conditions [25,26,27]. In fact, the progression of CVD is closely followed by or is accompanied by increasing obesity (especially visceral obesity), which means that the rate of occurrence of CVD may not decrease if the rate of obesity continues to increase [31,32].

In this study, obesity was induced in a rat model using a HF diet, which is similar to obesity in humans. Then, the potential effects of *N*-caffeoyltryptamine on plasma MCP-1 were mainly investigated because the chemokine is used as a common biomarker for CVD including atherosclerosis. In this study, the 16-week supplementation led to a significant decrease in plasma MCP-1 levels in the rats fed the HFS diet compared to those fed the HF diet. These data suggest that *N*-caffeoyltryptamine supplementation may specifically lower the plasma levels of the MCP-1 expression in rats fed a high-fat diet. This is also supported by the data that *N*-caffeoyltryptamine did not induce a significant difference in the plasma levels of other CVD biomarkers such as C-reactive protein and sE-selectin. However, one of the promising outcomes of this study was that the mean values of CRP and sE-selectin were lower in the HFS group than in the HF group, although this difference was not statistically significant. Therefore, it is possible that *N*-caffeoyltryptamine could lower plasma CRP and sE-selectin levels in a statistically significant manner when the supplement was given at higher doses and for a longer duration in the animal study. In this study, plasma levels of adiponectin were also confirmed higher in the HFS group than in the HF group, which was reported in our previous study [18]. As is well known, adiponectin is an adipose-tissue-derived hormone with anti-diabetic, anti-atherogenic and anti-inflammatory functions and is believed to play important roles in energy homoeostasis [18]. However, there is little information about the potential interactive effects of MCP-1 and adiponectin, especially with *N*-caffeoyltryptamine related to CVD. Therefore, it is currently planned to explore potential synergistic effects in a future study.

## 4. Materials and Methods

### 4.1. Materials

Tryptamine, caffeic acid, and other chemicals were purchased from Sigma Chemical Co. (St. Louis, MO, USA). *N*-caffeoyltryptamine was synthesized as described previously [17]. Monocytic THP-1 cells were obtained from the ATCC (Manassas, VA, USA). RPMI 1640 medium, fetal bovine serum (FBS), penicillin, and streptomycin were purchased from GIBCO (BRL Life Technologies, Grand Island, NY, USA).

### 4.2. p38 Kinase Activity Assays

Measurements of p38 activity were performed with the kinase selectivity profiling system CMGC-1 (Promega). This assay system kit is designed to screen potential inhibitors for p38 and other kinases in the CMGC Kinase Family. This kit utilizes a non-isotopic, sensitive, and specific method to measure the activities of p38 MAP kinase α, β, γ, and δ. Briefly, kinase working stock, ATP, and substrate working stock solutions were prepared by adding 2.5× kinase buffer to the kinase strips and 100 μM ATP to the substrate strips, respectively. Then, 2 µL kinase solution, 1 µL tested compounds, and 2 µL substrate solution were added to the wells. After that, the reaction was incubated for 60 min and ADP-Glo™ Kinase Assay was performed using a luminometer according to manufacturer’s protocol. The percent activities of p38 kinase activities (α, β, γ, and δ) were calculated in the presence of safflomide, inhibition curves were fit to a sigmoidal dose-response (variable slope) equation, and IC_50_ values were calculated using SigmaPlot 11.0 (Chicago, IL, USA) software.

### 4.3. Cell Culture

Monocytic THP-1 cells were grown in RPMI 1640 medium with 10% FBS, 100 units/mL penicillin, and 100 units/mL streptomycin. THP-1 cells were maintained at 37 °C in a humidified atmosphere with 5% CO_2_. For the MCP-1 expression, the cells (2 × 10^8^) were incubated for 15 min with several concentrations of *N*-caffeoyltryptamine (0, 10, 20, 40 µM) and then treated with LPS (0.01 µg/mL) for 16 h. Cell culture media was saved by centrifugation (3000 rpm, 10 min) and stored at −80 °C for MCP-1 assay.

### 4.4. Real Time RT-PCR Quantization of MCP-1 mRNA

From the LPS-stimulated THP-1cells, total RNA was isolated using TRIzol™ reagent (Invitrogen, Carlsbad, CA, USA), and 1 µg of total RNA was used to generate complementary DNA (cDNA) using AffinityScript Multiple Temperature cDNA Synthesis Kit (Agilent Technologies, Inc., Santa Clara, CA, USA). Real time semi-quantitative polymerase chain reaction (PCR) was carried out using TaqMan^®^ Fast Universal PCR Master Mix (2×) (Applied Biosystems, Foster City, CA, USA) and ViiA7 Real-Time PCR Detection System (Applied Biosystems). Human TaqMan probes and primers were purchased from Applied Biosystems: MCP-1/CCL2 (assay ID: Hs00234140_m) [18].

### 4.5. Determination of MCP-1 Protein in Cell Culture Medium

The saved samples were quickly thawed in a water bath (37 °C), and MCP-1 levels in the samples were measured using a Human CCL2/MCP-1 Quantikine ELISA Kit from R&D Systems (Minneapolis, MN, USA) according to manufacturer’s protocol.

### 4.6. Animal Study

Eight-week-old Sprague-Dawley male rats were purchased from Charles River (Wilmington, MA, USA). Rats were acclimated to the experimental facility for one week and housed in ventilated micro-isolator racks with an automatic watering system in a room with a 12:12-h light–dark cycle, ambient temperature of 18–23 °C, and relative humidity of 55.5%. All animal procedures were performed according to the animal protocol (BAACUC 13-025) approved on 15 December 2013 by the Beltsville Area Animal Care and Use Committee. Rats were fed an AIN-76A purified diet, which provided the recommended allowance of all nutrients required to maintain optimal health. For the study, rats were divided into three groups of eight animals each: control diet (C), high-fructose and fat diet (HF) and high-fructose and fat diet supplemented with *N*-caffeoyltryptamine in drinking water (2 mg per a day) (HFS), since our preliminary data suggested that C_max_ could be between 9 and 36 µM in rat plasma, if provided about 2 mg to 500 g rats (Avg. daily water consumption about 30 mL and the amount of *N*-caffeoyltryptamine about 6.6 mg per 100 mL drinking water). During the experiment, water bottles were individually set up in each rack to monitor the amounts of consumed water. Especially, the water consumption of the supplement group was closely monitored to provide about 2 mg of *N*-caffeoyltryptamine daily to the rats. Rats were fed the three diets for 16 weeks. Food and water intake was measured daily, and body weight was measured weekly. At the end of the experiment, animals were sacrificed following a 12-h fast, and blood was collected into EDTA-coated vials, centrifuged (3000 rpm for 10 min), and then the separated plasma was stored at −80 °C for further analysis.

### 4.7. Assays for Plasma Cholesterol, high-density lipoprotein cholesterol and Triglyceride

Plasma total cholesterol (TC), high-density lipoprotein cholesterol (HDL), and triglyceride (TG) were measured using a cholesterol assay kit (Cayman Chemical Inc., Ann Arbor, MI, USA), a HDL and LDL/VLDL cholesterol assay kit (Abcam, Cambridge, MA, USA), and a serum triglyceride determination kit (Sigma), respectively. Low-density lipoprotein cholesterol (LDL) was calculated from TG, TC, and HDL concentrations using the modified Friedwald formula: LDL = TC − HDL − 0.16 × (TG).

### 4.8. Assays for Plasma MCP-1, C-Reactive Protein and sE-Selectin

The measurements of MCP-1, C-reactive protein and sE-selectin were performed using plasma samples prepared from the animal study. Plasma MCP-1 levels were measured using the CCL2/MCP-1 Quantikine ELISA Kit from R&D Systems, C-reactive protein (CRP) was measured with the CRP ELISA Kit (Abcam, Cambridge, MA, USA), and sE-selectin levels were measured with the sE-Selectin/CD62E ELISA Kit (R&D Systems) according the manufacturers’ protocols.

### 4.9. Statistical Analysis

SigmaPlot 11.0 software was used for the statistical analysis. Multiple group data were analyzed using ANOVA followed by post-hoc analysis with the Bonferroni test. The unpaired Student’s *t*-test was used to compare means between the two groups. Values were considered significant when *p* < 0.05. Statistically significant differences are defined at the 95% confidence interval. Data are shown as mean ± SD.

## 5. Conclusions

MCP-1 is a well-known chemokine critically involved in the pathophysiological progression of several inflammatory diseases such as arthrosclerosis. *N*-caffeoyltryptamine is a phenolic amide with strong anti-inflammatory effects. Therefore, in this paper, the potential effect of *N*-caffeoyltryptamine on MCP-1 expression was investigated as a potential p38 MAP kinase inhibitor in vitro and in vivo. At the concentration of 20 μM, *N*-caffeoyltryptamine significantly inhibited p38 MAP kinase α, β, γ and δ, particularly p38 MAP kinase α (IC_50_ = 16.2 μM) and β (IC_50_ = 18.3 μM). The pretreatment of the THP-1 cells with *N*-caffeoyltryptamine (10, 20 and 40 μM) also led to significant suppression of MCP-1 mRNA and protein production by 10–45% (*p* < 0.05) in the cells. In the animal study, the average MCP-1 levels of the HF group were found to be higher than those of the C group (*p* < 0.05). However, the MCP-1 levels of the HFS group were significantly lower than those of the HF group (*p* < 0.05), suggesting that *N*-caffeoyltryptamine may decrease MCP-1 expression in vivo. However, there was no significant difference in the levels of other atherogenic markers, such as C-reactive protein and sE-selectin, between the HFS and HF groups, suggesting that *N*-caffeoyltryptamine may specifically suppress MCP-1 expression in vitro and in vivo, possibly by inhibiting p38 MAP kinase.

## Figures and Tables

**Table 1:** Composition of the control and high-fat diets. Diet value is in grams/kg. C, normal control diet; HF, high-fat diet; HFS, high-fat diet supplemented with *N*-caffeoyltryptamine in drinking water (2 mg per day).

Composition/Calories	C	HF	HFS
Casein	207	207	207
Cornstarch	400	400	400
Dyetrose	200	—	—
Cellulose	80	80	80
Lard	50	250	250
dl-Methionine	3	3	3
AIN-93 mineral mixture	50	50	50
AIN-93 vitamin mixture	10	10	10
*N*-Caffeoyltryptamine	—	—	2 mg/day
Calories (kcal/g)	3.678	4.678	4.678

In control diet, 3678 kcal/1000 g; 828 kcal (22.5%) from protein (casein); 2400 kcal (65%) from glucose and cornstarch; 450 kcal from fat (12.2%) (lard); In the HF diet, 4678 kcal/1000 g; 828 kcal (16.9%) from protein (casein); 1600 kcal (36.9%) from cornstarch; 2250 kcal from fat (46.1%) (lard).

**Table 2:** Effects of *N*-caffeoyltryptamine on diet intake, body weight gain, and calorie intake. C, normal control diet; HF, high-fat diet; HFS, high-fat diet supplemented with *N*-caffeoyltryptamine (2 mg per day). Data are presented as the mean ± SD (*n* = 8).

Diet and Calorie Intake/Bodyweight	C	HF	HFS
Diet intake (g/day)	23.2 ± 3.7	24.1 ± 4.1	23.9 ± 3.6
Initial body weight (g)	311 ± 39	306 ± 42	312 ± 41
Final body weight (g)	617 ± 51	659 ± 45	642 ± 41
Calorie intake (kcal/day)	85.3 ± 9.1	112.7 ± 19.1	111.8 ± 9.2

**Table 3:** Effects of *N*-caffeoyltryptamine on plasma lipid profiles. TG (triglyceride), Total-C (total cholesterol), HDL-C (high-density lipoprotein cholesterol) and LDL-C (low-density lipoprotein). C, normal control diet; HF, high-fat diet; HFS, high-fat diet supplemented with *N*-caffeoyltryptamine (2 mg per day). Data are presented as the mean ± SD (*n* = 8).

Lipid Profiles	C	HF	HFS
TG (mg/dL)	110 ± 18.3	131 ± 17.3	127 ± 19.2
Total-C (mg/dL)	80 ± 6.4	82 ± 7.8	81 ± 6.7
HDL-C (mg/dL)	51 ± 8.8	50 ± 8.7	49 ± 7.8
LDL-C (mg/dL)	11 ± 1.9	12 ± 2.2	12 ± 2.1
